# Cervical cancer systemic inflammation score: a novel predictor of prognosis

**DOI:** 10.18632/oncotarget.7378

**Published:** 2016-02-14

**Authors:** Ru-ru Zheng, Min Huang, Chu Jin, Han-chu Wang, Jiang-tao Yu, Lin-chai Zeng, Fei-yun Zheng, Feng Lin

**Affiliations:** ^1^ The Department of Gynecology, First Affiliated Hospital of Wenzhou Medical University, Zhejiang, Wenzhou, PR China; ^2^ The Department of Information and Engineering, Wenzhou Medical University, Zhejiang, Wenzhou, PR China

**Keywords:** cervical cancer, platelet to lymphocyte ratio, albumin, overall survival, disease free survival

## Abstract

Inflammation contributes to development and progression in a variety of cancers, including cervical cancer. We developed a novel cervical cancer systemic inflammation score (CCSIS) based on the preoperative platelet-to-lymphocyte ratio (PLR) and serum albumin levels. A retrospective analysis of clinical data from 795 patients with operable cervical cancer was then conducted to investigate the prognostic value of CCSIS and its association with the patients' clinicopathological features, overall survival (OS), and disease-free survival (DFS). CCSIS was predictive of OS and DFS. High CCSIS was correlated with more advanced FIGO stages, poor tumor differentiation, and the presence of PLN and LVSI. Both albumin levels and the PLR were independent prognostic indicators for operable cervical cancer. The use of the CCSIS could improve risk stratification and traditional clinicopathological analysis in cervical cancer.

## INTRODUCTION

Emerging evidence suggests that inflammation plays a key role in the initiation and progression of various cancers, including cervical cancer. Cancer-related inflammation can influence tumor cell proliferation, invasion, metastasis, cell survival, and angiogenesis [1, 2]. Accordingly, inflammation-based prognostic indicators, such as the modified Glasgow Prognostic Score (mGPS) [3, 4], platelet to lymphocyte ratio (PLR) [5, 6], neutrophil to lymphocyte ratio (NLR) [5–7], albumin, and C-reactive protein (CRP) [8, 9] are prognostic factors for cervical cancer as well as other cancers. These cost-effective biomarkers are used routinely in clinical settings and could be used to provide information regarding patient outcomes. However, there are few studies investigating the value of these biomarkers as independent indicators in cervical cancer. Additionally, to the best of our knowledge, integration of individual biomarkers to enhance the outcome prediction for cervical cancer has not yet been studied.

In this study, the prognostic values of PLR and albumin were investigated. We also developed a novel prognostic score, the cervical cancer systemic inflammation score (CCSIS), based on preoperative serum albumin levels and PLR. The correlation between CCSIS and clinicopathological parameters and the prognostic value of CCSIS in cervical cancer patients were investigated.

## RESULTS

### Patient characteristics

795 patients with clinically and histologically confirmed cervical cancer were included in the present analysis. These patients were diagnosed at a mean age of 49.5 ± 10.7 years, with a mean follow-up period of 62.3 ± 26.7 months. By the last follow-up, 123 patients had relapsed, and 98 patients had died. 105 (13.1%) of these cases were FIGO stage IA, 358 (44.9%) were IB, and the remaining 332 (42.0%) were IIA.

### Prognostic significance of variables and cutoff value determination

Cox proportional hazard models were used to identify associations between biomarkers and OS and DFS in the study population. As shown in Table 1, PLR and serum albumin were significant predictors of OS (*p =* 0.007 and *p* = 0.001, respectively), DFS (*p =* 0.004 and *p* = 0.001, respectively), FIGO stage, tumor differentiation, PLN, and LVSI (*p <* 0.05 for all) in cervical cancer patients, but not of NLR, age at diagnosis, parturition number, or histological subtype (*p >* 0.05 for all). In addition, LMR was an independent predictor of DFS (*p =* 0.037), but not OS (*p =* 0.063).

**Table 1:** Univariate Cox proportional hazards regression models of prognostic factors associated with overall survival (OS) and disease-free survival (DFS) in cervical cancer patients

Variable		OS		DFS	
		HR (95%CI)	*P*-value	HR (95%CI)	*p*-value
Parturition		1.369 (0.917-2.045)	0.124	1.412 (0.946-2.107)	0.091
Age	<48	1		1	
	≥48	1.090 (0.733-1.619)	0.671	1.097 (0.738-1.630)	0.648
NLR	<2.77	1		1	
	≥2.77	1.480 (0.995-2.201)	0.053	1.481 (0.997-2.201)	0.052
PLR	<128.3	1		1	
	≥128.3	1.746 (1.165-2.617)	0.007	1.767 (1.179-2.649)	0.006
LMR	<2.41	1		1	
	≥2.41	0.631 (0.392-1.014)	0.057	0.617 (0.384-0.993)	0.047
Albumin	<43.65	1			
	≥43.65	0.519 (0.348-0.773)	0.001	0.511 (0.343-0.761)	0.001
FIGO Stage	IA	1		1	
	IB	3.807 (1.176-12.321)	0.026	3.918 (1.211-12.679)	0.023
	IIA	5.524 (1.727-17.665)	0.004	5.944 (1.859-19.000)	0.003
Differentiation	Well	1		1	
	Mod.	3.969 (1.429-11.028)	0.008	3.913 (1.409-10.872)	0.009
	Poorly	7.337 (2.644-20.357)	<0.001	7.268 (2.620-20.157)	<0.001
PLN	No	1		1	
	Yes	3.104 (2.025-4.759)	<0.001	3.259 (2.127-4.992)	<0.001
LVSI	No	1			
	Yes	1.821 (1.170-2.836)	0.008	1.887 (1.212-2.938)	0.005
Histological	Squ				
	Non-S	0.982 (0.600-1.607)	0.942	0.953 (0.583-1.559)	0.848
SIS	0	1			
	1	1.987 (1.153-3.423)	0.013	1.967 (1.142-3.386)	0.015
	2	3.187 (1.793-5.664)	<0.001	3.290 (1.852-5.843)	<0.001

PLR: platelet-lymphocyte ratio, NLR: neutrophil-lymphocyte ratio, LMR: lymphocyte-monocyte ratio, PLN: pelvic lymph node metastasis, LVSI: vascular lymph node invasion

A multivariate Cox proportional hazard analysis was used to evaluate the independent prognostic factors for OS and DFS. Both PLR and serum albumin were independent prognostic factors for OS (hazard ratio [HR] 1.547, 95% CI 1.028 to 2.328*, p* = 0.036; HR 0.619, 95% CI 0.412 to 0.930, *p* = 0.021, respectively) and DFS (HR 1.563, 95% CI 1.087 to 2.245, *p* = 0.016; HR 0.680, 95% CI 0.473 to 0.979, *p* = 0.038, respectively) (Tables 2 and 3).

According to the ROC curves, the best cutoff values for NLR, PLR, LMR, and albumin corresponding to maximum joint sensitivity and specificity were 2.77, 128.3, 2.41 and 43.65 g/L, respectively.

### Survival analysis of patients stratified according to cutoff values

**Figure 1:**
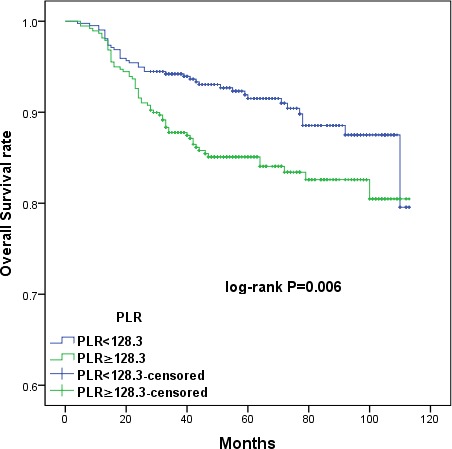
Kaplan-Meier survival curves showing the relationship between overall survival (OS) in cervical cancer patients and preoperative PLR Patients were stratified into high and low PLR groups using the cutoff value of 128.3; patients with higher PLR (PRL ≥ 128.3) had shorter OS (*p =* 0.006).

**Figure 2:**
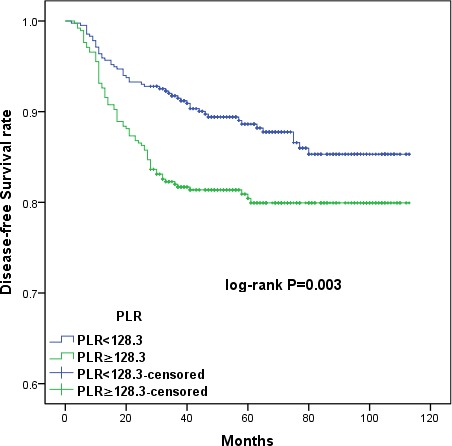
Kaplan-Meier survival curves showing the relationship between disease-free survival (DFS) in cervical cancer patients and preoperative PLR Patients with higher PLR (PRL ≥ 128.3) had shorter DFS (*p =* 0.003).

**Figure 3:**
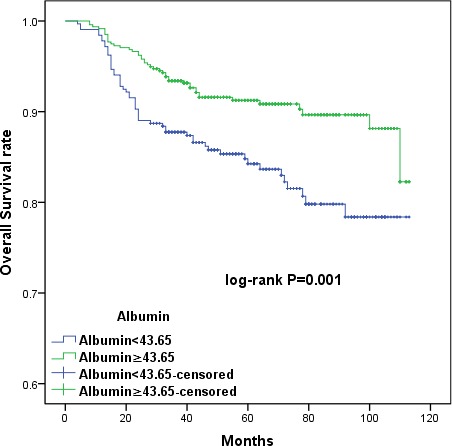
Kaplan-Meier survival curves showing the relationship between overall survival (OS) in cervical cancer patients and preoperative albumin levels Patients were stratified into high and low albumin groups using the cutoff value of 43.65. Patients with lower serum albumin levels (albumin < 43.65) had shorter OS (*p =* 0.001).

**Figure 4:**
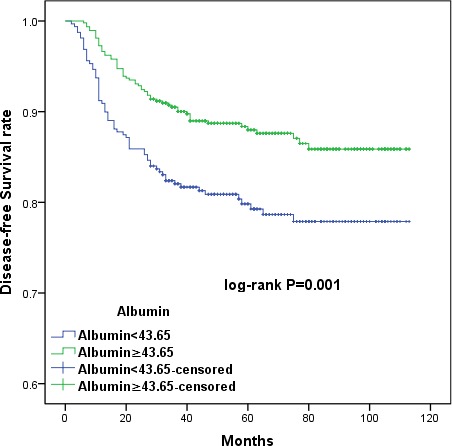
Kaplan-Meier survival curves showing the relationship between disease-free survival (DFS) in cervical cancer patients with cervical cancer and preoperative albumin levels Patients with lower serum albumin levels (albumin < 43.65) had shorter DFS (*p =* 0.001).

The large patient cohort was divided into high- and low-PLR (< 128.3 *vs*. ≥ 128.3) and albumin (< 43.65g/L *vs*. ≥ 43.65g/L) groups using the cut-off values. Kaplan-Meier survival curves demonstrated that increased PLR and decreased serum albumin were associated with shorter OS (*p =* 0.006 and *p* = 0.001, respectively) and DFS (*p =* 0.003 and *p* = 0.001, respectively) (Figures 1, 2, 3, and 4). Cumulative 3- and 5-year OS (94.2% and 92.3%) and DFS (91.8% and 89.4%) rates in the low-PLR group were higher than OS (87.9% and 85.6%) and DFS (82.1% and 81.0%) rates in the high-PLR group (OS *p* = 0.002 and *p* = 0.003, respectively; DFS *p* < 0.001 and *p* = 0.001, respectively). Patients with low albumin had shorter 3- and 5-year OS (91.8% and 89.4%) and DFS (82.1% and 80.6%) than the high albumin group (OS 93.5% and 91.8%, *p* = 0.005 and *p* = 0.004, respectively; DFS 90.5% and 88.7%, *p* = 0.001 and *p* = 0.002, respectively).

### Combining PLR and albumin to provide additional stratification

**Figure 5:**
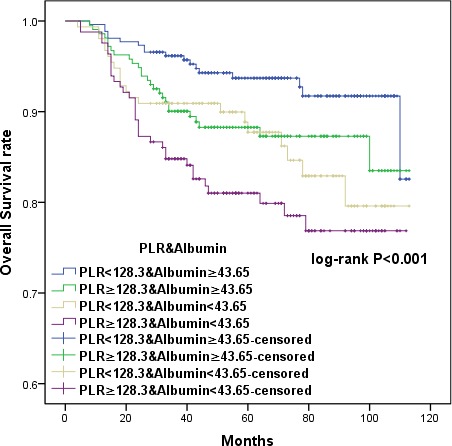
Kaplan-Meier survival curves showing the relationship between overall survival (OS) in cervical cancer patients and the combination of preoperative PLR and albumin Patients were separated into four groups as follows: PLR < 128.3 and albumin ≥ 43.65; PRL ≥ 128.3 and albumin ≥ 43.65; PLR < 128.3 and albumin < 43.65; and PRL ≥ 128.3 and albumin < 43.65. Patients with either serum albumin < 43.65 or PLR ≥ 128.3 had similar OS *(p =* 0.697).

**Figure 6:**
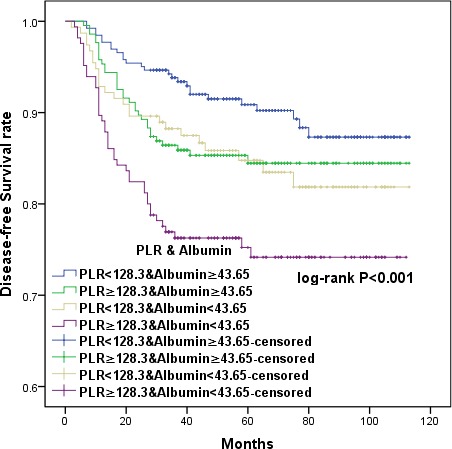
Kaplan-Meier survival curves showing the relationship between disease-free survival (DFS) in cervical cancer patients with cervical cancer and the combination of preoperative PLR and albumin Patients with either serum albumin < 43.65 or PLR ≥ 128.3 had similar DFS (*p =* 0.872).

To further distinguish patients with different oncological outcomes, we defined four subgroups by combining PLR with serum albumin levels. OS and DFS were similar in subgroups with serum albumin < 43.65 or PLR ≥ 128.3 (HR 1.121, 95% CI 0.631 to 1.993, *p* = 0.697; HR 1.045, 95% CI 0.615-1.773, *p* = 0.872, respectively) (Figures 5 and 6). Therefore, we combined these two subgroups to create three CCSIS groups defined as follows: patients with both increased PLR and decreased serum albumin (PLR ≥ 128.3 and albumin < 43.65 g/L) were assigned a score of 2; patients with either increased PLR or decreased serum albumin were assigned scores of 1; and patients with both decreased PLR and increased serum albumin (PLR < 128.3 and albumin ≥ 43.65 g/L) were assigned scores of 0.

**Figure 7:**
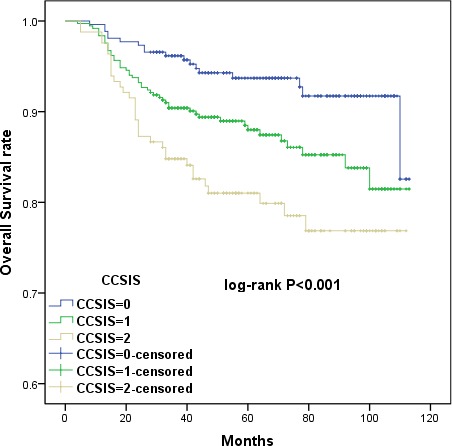
Kaplan-Meier curves showing the relationship between overall survival (OS) in cervical cancer patients and cervical cancer systemic inflammation scores (CCSIS) of either 0, 1, or 2 Higher CCSIS scores were associated with shorter OS (*p <* 0.001).

**Figure 8:**
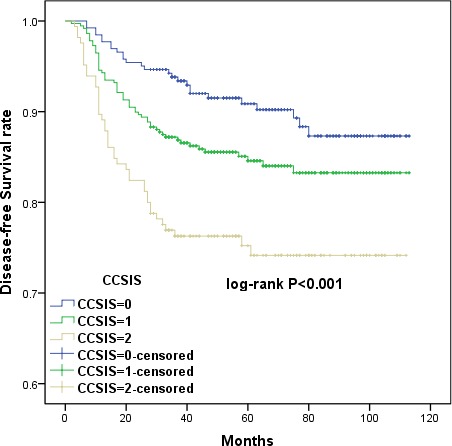
Kaplan-Meier curves showing the relationship between disease-free survival (DFS) in cervical cancer patients and CCSIS Higher CCSIS scores were associated with shorter DFS (*p <* 0.001).

Kaplan-Meier curves showed that high CCSIS scores were associated with shorter OS and DFS *(p <* 0.001 for both) (Figures 7 and 8). The cumulative 3-year OS rates were 84.8%, 90.4%, and 96.2% for patients with CCSIS scores of 2, 1, and 0, respectively; the 5-year OS rates in corresponding subgroups were 81.9%, 88.9% and 94.3%, respectively. The differences in cumulative OS among the three subgroups were statistically significant (*p <* 0.001 for both). Furthermore, the cumulative 3-year DFS rates were 76.4%, 87.2%, and 93.9% for patients with CCSIS scores of 2, 1, and 0, respectively; 5-year DFS rates in the corresponding subgroups were 75.8%, 85.3%, and 91.6 %. The differences among the three subgroups for cumulative DFS were also statistically significant (*p* < 0.001 for both).

CCSIS was predictive of prognosis as measured by both OS and DFS in a univariate analysis *(p <* 0.05 for both) (Table 1). In multivariate analysis, both CCSIS and tumor differentiation *(p <* 0.05 for all) were independent predictors of OS and DFS in cervical cancer patients (Tables 2 and 3). Furthermore, the presence of LVSI was associated with higher risk of recurrence and increased FIGO stage.

**Table 2:** Multivariate Cox proportional hazards regression models of prognostic factors associated with overall survival in cervical cancer patients

Variable		Multivariate^a^		Multivariate^b^	
		HR (95%CI)	*p*-value	HR (95%CI)	*p*-value
PLR	<128.3	1			
	≥128.3	1.544 (1.026-2.323)	0.037		
Albumin	<43.65	1			
	≥43.65	0.620 (0.413-0.932)	0.022		
FIGO Stage	IA & IB	1		1	
	IIA	1.522(1.014-2.285)	0.043	1.545 (1.029-2.319)	0.036
Differentiation	Well & Mod.	1	0.001	1	
	Poorly	1.970 (1.315-2.950)		1.984 (1.325-2.971)	0.001
LVSI	No	1		1	
	Yes	1.404 (0.889-2.218)	0.145	1.401 (0.888-2.210)	0.147
SIS	0			1	
	1			1.914 (1.110-3.299)	0.019
	2			2.601 (1.452-4.660)	0.001

a Adjustment for all variables listed in the table, except for CCSIS.

b Adjustment for all variables listed in the table, except for PLR and Albumin.

PLR: platelet-lymphocyte ratio

LVSI: vascular lymph node invasion

**Table 3:** Multivariate Cox proportional hazards regression models of prognostic factors associated with disease- free survival among patients with cervical cancer

Variable		Multivariate^a^		Multivariate^b^	
		HR (95%CI)	*p*-value	HR (95%CI)	*p*-value
PLR	<128.3	1			
	≥128.3	1.591 (1.058-2.392)	0.026		
LMR	<2.41	1			
	≥2.41	0.769 (0.473-1.249)	0.289		
Albumin	<43.65	1			
	≥43.65	0.612 (0.408-0.919)	0.018		
FIGO stage	IA & IB	1		1	
	IIA	1.610 (1.073-2.415)	0.021	1.627 (1.085-2.439)	0.019
Differentiation	Well & Mod.	1		1	
	Poorly	1.952 (1.305-2.920)	0.001	1.964 (1.313-2.938)	0.001
LVSI	No	1		1	
	Yes	1.417 (0.896-2.241)	0.136	1.415 (0.896-2.234)	0.137
SIS	0			1	
	1			1.897 (1.101-3.269)	0.021
	2			2.698 (1.508-4.828)	0.001

a Adjustment for all variables listed in the table, except for CCSIS.

b Adjustment for all variables listed in the table, except for PLR and Albumin.

PLR: platelet-lymphocyte ratio

LMR: lymphocyte-monocyte ratio

LVSI: vascular lymph node invasion

### Correlations between PLR, albumin, CCSIS, and other clinical parameters of cervical cancer

Clinicopathologic characteristics were compared between patients grouped by PLR and serum albumin as shown in Table 4. Increased PLR and decreased serum albumin were both associated with advanced FIGO stage (*p =* 0.040 and *p* < 0.001, respectively) and poor tumor differentiation (*p =* 0.010 and *p* < 0.001, respectively). Additionally, elevated PLR was associated with the presence of PLN (*p =* 0.002). In contrast, decreased serum albumin was associated with the presence of LVSI (*p <* 0.001). As shown in Table 5, high CCSIS score was correlated with advanced FIGO stage (*p <* 0.001), poor tumor differentiation (*p <* 0.001), and the presence of PLN (*p =* 0.002) and LVSI (*p =* 0.010).

**Table 4:** Baseline characteristics of cervical cancer patients stratified by platelet-lymphocyte ratio (PLR) and albumin

Variables	PLR<128.3	PLR≥128.3	*p*-value	Albumin<43.65	Albumin≥43.65	*p*-value
	(*N* = 416)	(*N* = 319)		(*N* = 319)	(*N* = 476)	
Age (years)	50.8±11.1	48.1±10.1	0.001	49.6±10.7	49.4±10.6	0.851
Parturition	2.9±1.6	2.6±1.4	0.010	2.8±1.5	2.7±1.5	0.363
FIGO stage			0.040			<0.001
IA	67	38		27	78	
IB	180	178		135	223	
IIA	169	163		157	175	
Differentiation			0.010			<0.001
Well	70	64		40	94	
Mod.	236	179		157	258	
Poorly	110	136		122	124	
PLN			0.002			0.051
No	374	312		266	420	
Yes	42	67		53	56	
LVSI			0.547			<0.001
No	347	310		244	413	
Yes	69	69		75	63	
Histological subtype			0.297			0.018
Squamous	330	289		262	357	
Non-squamous	86	90		57	119	

PLN: pelvic lymph node metastasis

LVSI: vascular lymph node invasion

**Table 5:** Baseline characteristics of cervical cancer patients stratified by cervical cancer systemic inflammation score (CCSIS) (*N*=795)

Variables	SIS = 0	SIS = 1	SIS = 2	*p*-value
	(*N* = 262)	(*N* = 368)	(*N* = 165)	
Age (years)	50.9±11.0	48.8±10.6	48.8±1.6	0.024
Parturition	2.9±1.6	2.7±1.4	2.7±1.6	0.054
FIGO stage				<0.001
IA	48	49	8	
IB	114	175	69	
IIA	100	144	88	
Differentiation				<0.001
Well	52	60	22	
Moderately	148	198	69	
Poorly	62	110	74	
PLN				0.002
No	239	316	131	
Yes	23	52	34	
LVSI				0.010
No	231	298	128	
Yes	31	70	37	
Histological subtype				0.166
Squamous	195	297	127	
Non-squamous	67	71	38	

PLN: pelvic lymph node metastasis

LVSI: vascular lymph node invasion

## DISCUSSION

The clinicopathological characteristics and prognoses of 795 cervical cancer patients were investigated in this retrospective study. Univariate Cox proportional hazard analysis revealed that PLR, serum albumin, FIGO stage, tumor differentiation, PLN, and LVSI, but not NLR, were associated with OS and DFS. Additionally, increased PLR and decreased serum albumin before surgery were independent predictors of shorter OS and DFS in a multivariate analysis. To find a more objective marker, the cervical cancer systemic inflammation score (CCSIS), an integrated indicator based on PLR and serum albumin, was created. Kaplan-Meier curves demonstrated that high CCSIS scores were associated with shorter OS and DFS. Furthermore, increased CCSIS was associated with advanced FIGO stage, poor tumor differentiation, and the presence of PLN and LVSI.

Platelets and lymphocytes are related to immune surveillance [10]. Therefore, PLR, a combined index of platelets and lymphocyte counts, has been investigated as a prognostic factor in various cancers [11]. Recently, a meta-analysis including 12,754 patients demonstrated that high PLR was associated with shorter OS in various solid tumors [12]. The prognostic value of PLR in cervical cancer is unclear. Mesut et al. found that PLR may provide useful information regarding cervical carcinoma invasion in a study of 75 patients with pre-invasive and 30 patients with invasive disease [13]. On the contrary, Wang et al. suggested that pretreatment PLR did not predict prognosis in cervical cancer patients. However, that study only included 111 FIGO stage IB2-IIB patients who had received neoadjuvant chemotherapy and underwent radical hysterectomy [14]. In a study of 460 FIGO stage I or II cervical cancer patients, Yu et al. found that NLR, but not PLR, was an independent prognostic marker for PFS, but not for OS [6]. However, unlike phosphoglycerate dehydrogenase [15], PLR is a cheap and easily available biomarker that may be an independent predictor of poor prognosis in patients with cervical cancer.

Serum albumin is a negative acute phase protein. It has been integrated with other markers to create new prognostic markers, such as mGPS [4]. Decreased preoperative albumin is associated with reduced survival and poor prognosis in other cancer patients [16–18]. However, few studies have investigated the relationship between preoperative albumin and patient survival in cervical cancer. In a study of 238 patients who underwent chemoradiotherapy instead of operations, mGPS was correlated with advanced stages in cervical cancer patients and was an independent prognostic indicator for OS and PFS [19]. Consistent with previous studies, we showed that decreased albumin was associated with poor OS and DFS in operable cervical cancer patients.

So far, no studies have explored the links between PLR and serum albumin in cervical cancer. Here, we demonstrated for the first time that decreased albumin predicted shorter OS and DFS. By creating a new score that combines PLR and albumin, we stratified patients into three groups. This approach was particularly successful in predicting differences among the three groups in OS and DFS in multivariate models. Moreover, subgroup analysis supported the validity of this stratification method. Therefore, PLR and albumin were useful prognostic biomarkers that, when combined, provided additional risk stratification for cervical cancer patients. Patients with high CCSIS scores, which were correlated with aggressive cancer biology phenotypes such as advanced FIGO stage, poor tumor differentiation, and the presence of PLN and LVSI, might benefit from aggressive therapeutic regimens such as radiotherapy and/or chemotherapy, or even neoadjuvant chemotherapy followed by surgery [20, 21].

These results reflect complex interactions between systematic inflammation and tumor progression. Both albumin and lymphocyte levels are indicative of host immunity. Albumin, platelets and lymphocytes play different roles in systematic inflammation which leads to tumor progression. Synthesized predominantly in the liver [22], albumin is a safe and immunogenic protein. Decreased preoperative albumin may indicate a malnourished state [23] and sustained systemic inflammation [24, 25], suggesting a suppressed immune system. Platelets are the major serum source of vascular endothelial growth factor (VEGF), which induces angiogenesis and endothelial proliferation [3, 26–28]. Angiogenesis in turn is critical for the growth and metastasis of tumors [29]. T lymphocytes are critical adaptive immune cells, which are commonly divided into two subsets based on the expression of CD4 and CD8 receptors [30]. On one hand, decreased lymphocyte levels can cause immune suppression, which can produce inflammatory cytokines in the tumor microenvironment [31]. On the other hand, according to Ramello et al, after co-incubation with CD4+ or CD8+ tumor-induced senescent T cells, monocytes produce more pro-inflammatory cytokines (TNF, IL-1β and IL-6) and angiogenic factors (MMP-9, VEGF-A and IL-8) than those co-cultured with controls [32]. Thus, T cells may play key role in angiogenesis and impact tumor progression and metastasis. Overall, PLR may reflect the balance between tumor angiogenesis and host immunity. Increased PLR indicates a predominance of pro-tumor angiogenesis responses and is associated with poor oncologic outcomes. Increased PLR together with decreased albumin, which indicates hypoimmunity in patients, may lead to cancer. The present study supports this theory.

Currently, this is the largest study of the prognostic roles of preoperative PLR and albumin in cervical cancer. It is also the first study to propose a potential prognostic role for CCSIS for this disease. However, the present study does have some limitations. First, it is difficult to control for potential confounding factors in retrospective studies, and the results might differ in other populations; future studies replicating these findings in diverse patient groups are needed. Second, the present study was conducted at a single institution, and multicenter studies of the markers used here would strengthen our conclusions. Finally, only patients who underwent surgery were included, and the results may not apply to patients without surgical indications, including stages IIB, III and IV.

In conclusion, this study highlights the potential role of PLR and albumin as prognostic factors for OS and DFS in early-stage cervical cancer patients. CCSIS, a new biomarker integrating PLR with serum albumin levels, was evaluated. CCSIS served as an independent prognostic factor and might aid in the identification of cervical cancer patients and in managing their treatment.

## MATERIALS AND METHODS

### Patients

The current analysis included 795 patients with cervical cancer who received treatment at the First Affiliated Hospital of Wenzhou Medical University (Zhejiang province, China) between May 2005 and December 2012. Only patients with IA1-IIA2 stage cervical cancer who had received radical hysterectomy with additional chemotherapy or radiotherapy at the hospital were included. Patients with any history of immunological, hematological, or liver disease, other cancers, adolescents (< 18 years old), and patients who took medications that could cause hematological disease or liver disease were excluded. This study was approved by the Hospital Ethics Committee of the First Affiliated Hospital of Wenzhou Medical University. As this was a retrospective study, written consents were waived, and verbal consents were obtained *via* telephone.

### Data collection and definitions of NLR, PLR and LMR

Albumin levels and preoperative blood cell counts, which included platelet, absolute lymphocyte, neutrophil, and monocyte levels, from one week prior to operation were collected through the electronic medical record system (EMRS). NLR, PLR, and lymphocyte to monocyte ratio (LMR) were calculated as absolute neutrophil count divided by absolute lymphocyte count, absolute platelet count divided by absolute lymphocyte count, and absolute lymphocyte count divided by absolute monocyte count, respectively. Patient clinical and pathological characteristics, such as age at diagnosis, parturition number, FIGO stage, pathological types, tumor differentiation, vascular lymph node invasion (LVSI), and pelvic lymph node metastasis (PLN) were also collected.

### Follow-up and prognosis evaluation

Out-patient medical records, telephone consultations, and social security death indices were used to calculate overall survival (OS) and disease free survival (DFS). Follow-ups were conducted every 3 months for the first 2 years, 6 months for the next 3 years, and annually in the following years. Gynecological examinations, cervical cytology, trans-vaginal ultrasound scanning, and computed tomography (CT) and/or magnetic resonance imaging (MRI) were conducted during follow-up evaluations. OS was defined as the time from surgery to the time of death regardless of cause. DFS was calculated from the date of surgery to the date of recurrence.

### Statistical analysis

Continuous variables with normal distributions are expressed as mean ± standard deviation (SD) and were compared using standard *t*-tests. Wilcoxon's rank-sum test or the Kruskal-Wallis test were used for continuous variables with non-normal distributions. Additionally, the Chi-square test or Fisher's exact test were applied to analyze categorical variables. Survival analyses were performed using Kaplan-Meier survival curves, and significant differences between groups were compared by the log-rank test. Cox proportional hazard regression was used to identify associations between outcomes and variables. Variables identified as statistically significant in the univariate Cox regression analysis were then used in multivariate analysis with backward stepwise selection. Threshold values for NLR, PLR, LMR, and serum albumin were selected using receiver operating characteristic (ROC) curve analysis.

NLR, PLR, LMR and serum albumin biomarkers were evaluated together with traditional clinicopathological variables in univariate and multivariate analyses. PLR and serum albumin were identified as independent prognostic indicators for OS and DFS. The CCSIS combined PLR and serum albumin levels and was assessed in a multivariate analysis with traditional clinicopathological variables. All *p* values were 2-sided, and a *p* value < 0.05 was considered statistically significant. Statistical analysis was performed using the Statistical Package for Social Sciences version 21.0 (SPSS Inc., Chicago, IL, USA).
